# Enhanced location tracking in sensor fusion-assisted virtual reality micro-manipulation environments

**DOI:** 10.1371/journal.pone.0261933

**Published:** 2021-12-28

**Authors:** John David Prieto Prada, Jintaek Im, Hyondong Oh, Cheol Song

**Affiliations:** 1 Department of Robotics Engineering, DGIST, Daegu, South Korea; 2 School of Mechanical, Aerospace and Nuclear Engineering, UNIST, Ulsan, South Korea; Boston University, UNITED STATES

## Abstract

Virtual reality (VR) technology plays a significant role in many biomedical applications. These VR scenarios increase the valuable experience of tasks requiring great accuracy with human subjects. Unfortunately, commercial VR controllers have large positioning errors in a micro-manipulation task. Here, we propose a VR-based framework along with a sensor fusion algorithm to improve the microposition tracking performance of a microsurgical tool. To the best of our knowledge, this is the first application of Kalman filter in a millimeter scale VR environment, by using the position data between the VR controller and an inertial measuring device. This study builds and tests two cases: (1) without sensor fusion tracking and (2) location tracking with active sensor fusion. The static and dynamic experiments demonstrate that the Kalman filter can provide greater precision during micro-manipulation in small scale VR scenarios.

## Introduction

VR applications are commonly applied to various simulators with large scale scenarios. Currently, VR has been investigated for managing stress caused by anxiety disorders [1], navigation and tracking systems [2], minimizing hand tremor [3], and in medical simulators that teach subjects how to use surgical tools through realistic scenarios in virtual environments [4]. A simulator used the MeVisLab platform merged with the HTC headset to interact fast and instantly in medical scenarios [5]. Its medical data enabled an immersive environment through the HTC headset. This VR environment allows individuals to gain valuable knowledge on critical tasks. Furthermore, VR environments can immerse the subjects to provide auto-control of the experiment [6]. For example, in large scale VR environments, high levels of immersion and presence can lead to behavioral realism [7].

Recent VR studies implemented sensor fusion techniques to enhance the performance of VR system mainly in large scale scenarios. For example, one study combined multiple visual servoing with 3-D VR-based simulation techniques for a micro-assembly task [8]. The implementation of two different techniques such as visual force/position servoing and real-time 3-D computer graphics VR enables the integration of efficient micro-assembly workcells processes. In addition, Song designed a methodology to predict VR-controller positions in real-time [9]. This work demonstrated an accurate tracking of controllers with estimation errors up to 2 cm. Similarly, Tannus and Naves used low-cost inertial measurement units (IMUs) for motion tracking in VR environments [10]. This study compared several sensor fusion algorithms for positioning prediction. Various methods for enhancing the position in VR have been widely investigated, mainly in large-scale scenarios. In general, most of works used several and bulky array sensors in both handheld devices and HMD. Until now, there has been growing a research interest in introducing sensor fusion algorithms and predicting the VR position tracking in large scale scenarios.

To apply the VR technology into microsurgery and microassembly, the positioning performance of commercial VR controllers should be enhanced particularly in small scale scenarios in the order of millimeters. The commercial VR systems often limit users to either finger’s motion or handheld devices for virtual interactions [11–13]. A study demonstrated that the accuracy of the Oculus Touch VR-controller has a large error in a relatively small-scale step-size [14]. This study determined the relative position accuracy of the Oculus Touch controllers in a 2.4 m × 2.4 m area. The greatest inaccuracy was 12.7 ± 9.9% at step size of 6.23 mm. Thus, the current commercial VR controllers lack the precision in a millimeter scale VR environment for vitreoretinal microsurgical training.

The purpose of this study is to enhance the VR controller tracking system by using sensor fusion in a millimeter situation. To increase the tracking performance of VR controller, we introduced the KF as a sensor fusion technique without destroying commercial VR controller. The KF algorithm was implemented between the VR controller and the IMU. The presented system comprises a commercial VR controller, an inertial measuring unit (IMU) sensor, and microsurgical tool such as forceps. The millimeter scale VR scenario displays a simulated image of forceps in virtual space. Fifteen subjects followed a given path that was provided in two different cases: printed on paper and visualized in the VR space. Also, we compared the conditions when KF was activated and deactivated within the situations. The stationary and dynamic experimental results showed improved position accuracy in situations of micro-localization when the KF algorithm was enabled.

## Implementation of the VR system

### System architecture

**Fig 1:**
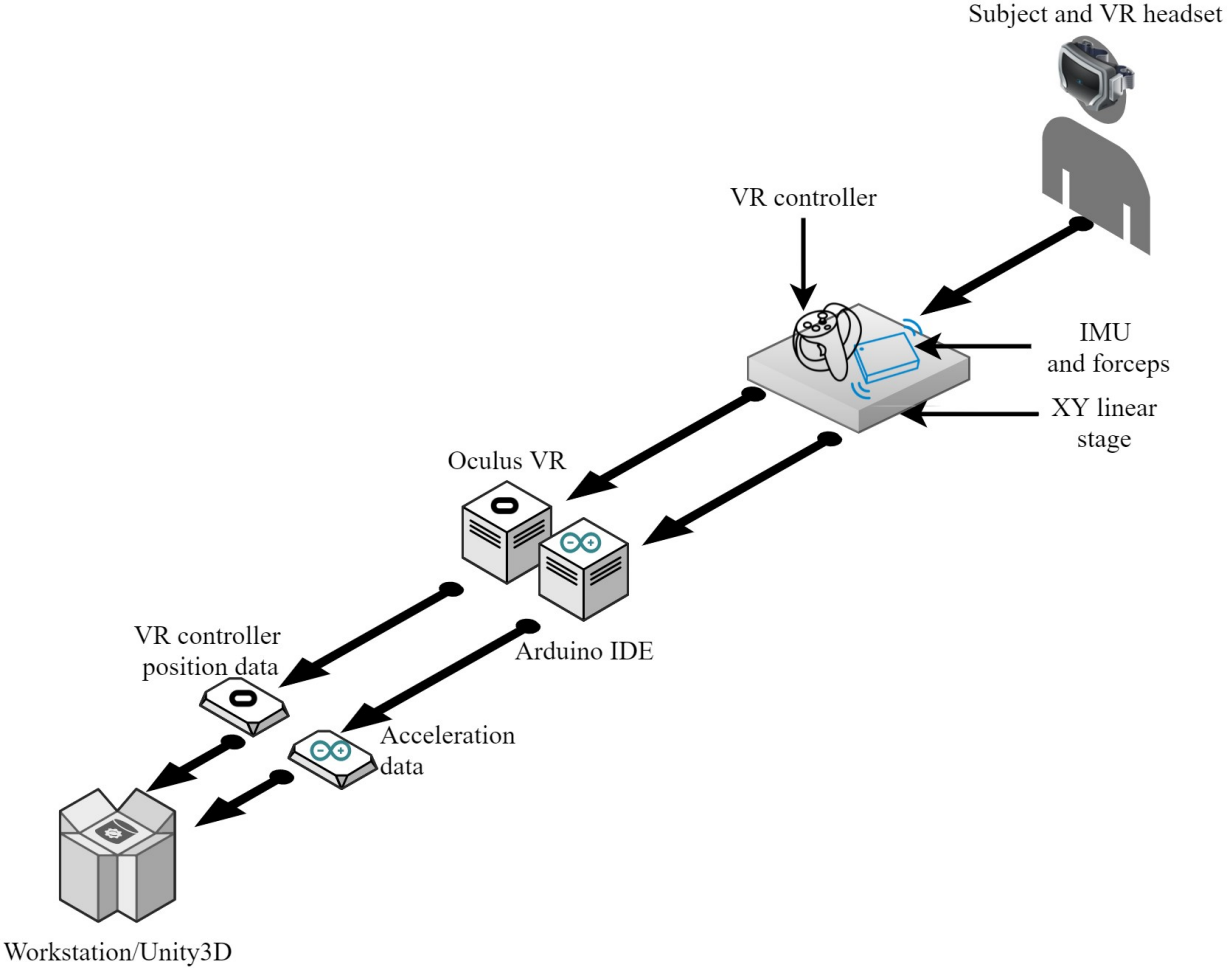
Architecture of the system. The subject wears a VR headset and moves a linear stage along the X and Y axes. The VR controller, IMU, and forceps are attached to a linear stage. Acceleration data is processed via the Arduino integrated development environment (IDE) software and then sent to Unity software. The VR controller data is acquired by Unity software as well.

The system consists of two distinct parts: the hardware and the software. Fig 1 shows the architecture of the system as a whole. The subject wears the head-mounted display (HMD) and grabs the forceps to conduct the experiments. A proper real-time simulation can be performed by having VR equipment such as an HMD, a VR controller, and Arduino connected to the workstation. The external electronic components are the IMU sensor with 9 degrees of freedom (DOF), the VR controller, and forceps. The components mounted on the single-piece hardware send the acceleration data to the Arduino board. Next, the Arduino board processes and sends IMU data to the Unity VR system. The location data from the VR controller is integrated into the KF algorithm. The VR system has an experimental space of 12 mm × 12 mm, a field of view of 110 degrees, and a resolution of 1080 × 1200 pixels per eye.

### Hardware system

**Fig 2:**
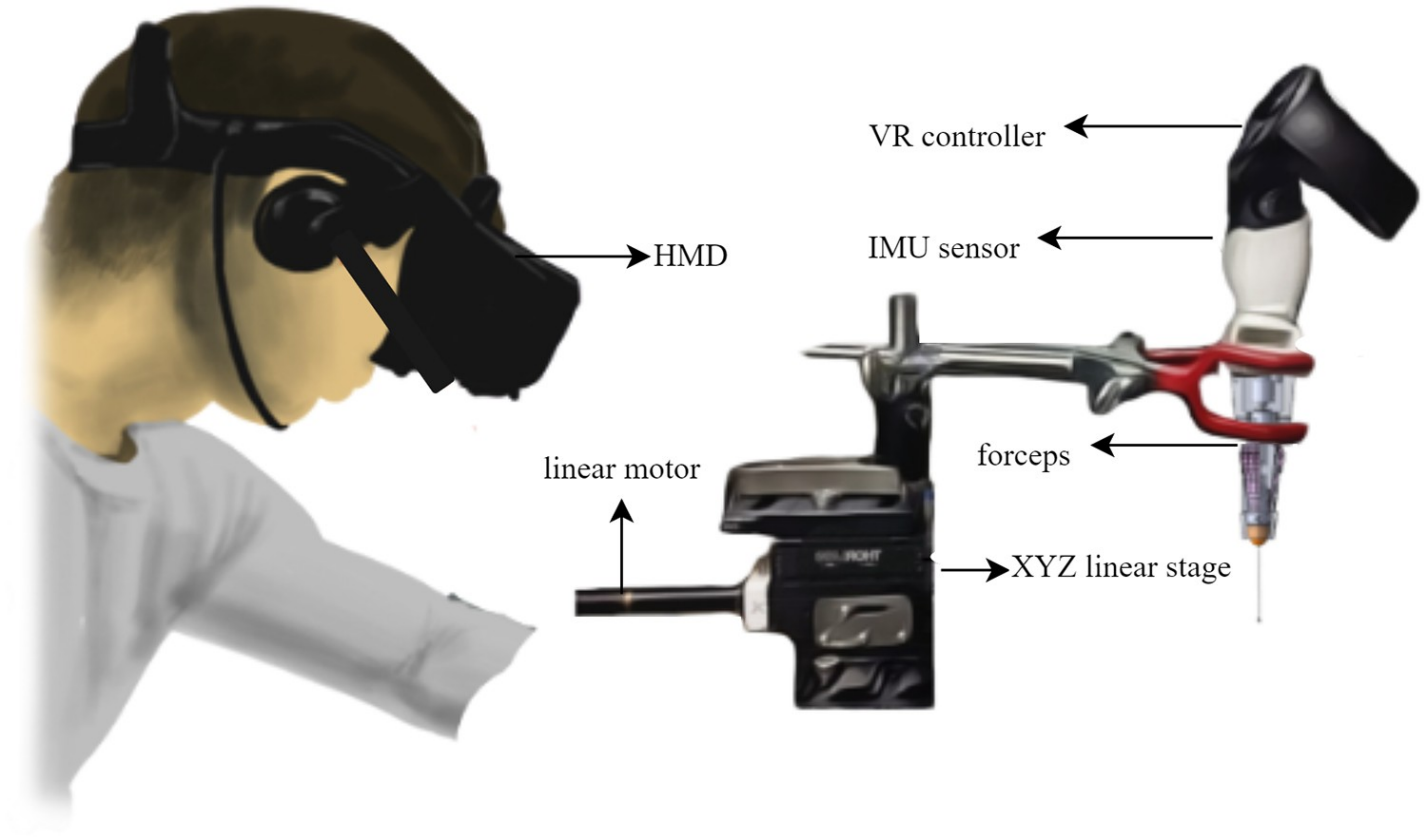
Hardware architecture. The hardware is composed of a HMD, an IMU sensor, a VR controller, forceps, and an XY linear stage.

Fig 2 shows the hardware components of the system. It consists of a 2-D position controller, an XY linear stage, forceps, and an IMU sensor attached to the controller in a single-piece structure. For the simplicity of 2-D traces, all the measurement systems and forceps are mounted on the XY linear stage. The Arduino reads the data from IMU and the XY linear stage enables the free movement of the single-piece structure for controlled experiments. The XY linear stage was used in two ways during experiments: hand motion actions and motorized tracing.

### Software system

**Fig 3:**
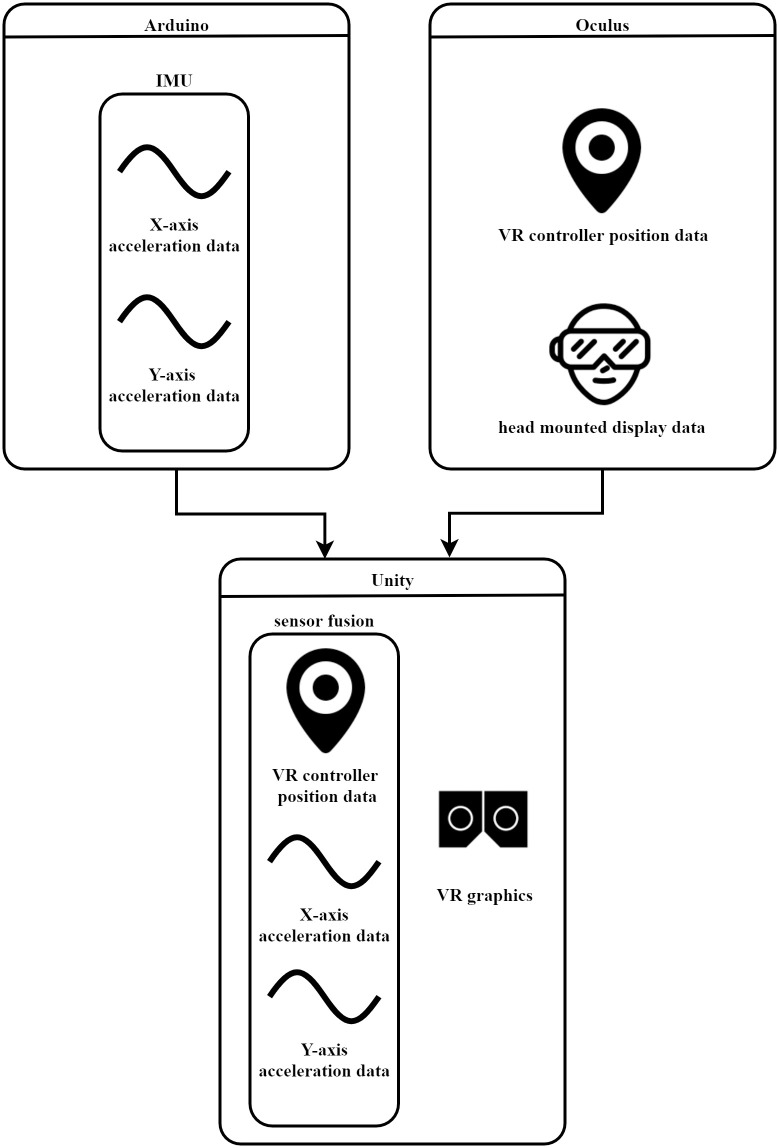
Software system. Arduino IDE, Oculus SDK, and Unity were the software used in this study. Arduino processes all the electronic signals provided by the IMU. Oculus gathers the VR signals. Unity merges the data from the IMU and VR controller in a sensor fusion algorithm. The VR graphics are processed in Unity as well.

Fig 3 shows the software used in this study. The software system was designed in various programming languages. For the electronics part, the IMU sensor sends the acceleration data to the Arduino board via an I2C communication protocol based on the C/C++ language. The data from the IMU is sent via the Arduino board. C#-based Unity software receives and processes IMU data via the RS232 communication protocol from the Arduino board. Both the graphics and VR environments were processed using Unity software. Thus, the HMD graphics come directly from Unity VR scenario.

## Implementation of the sensor fusion algorithm

### Kalman filter

**Fig 4:**
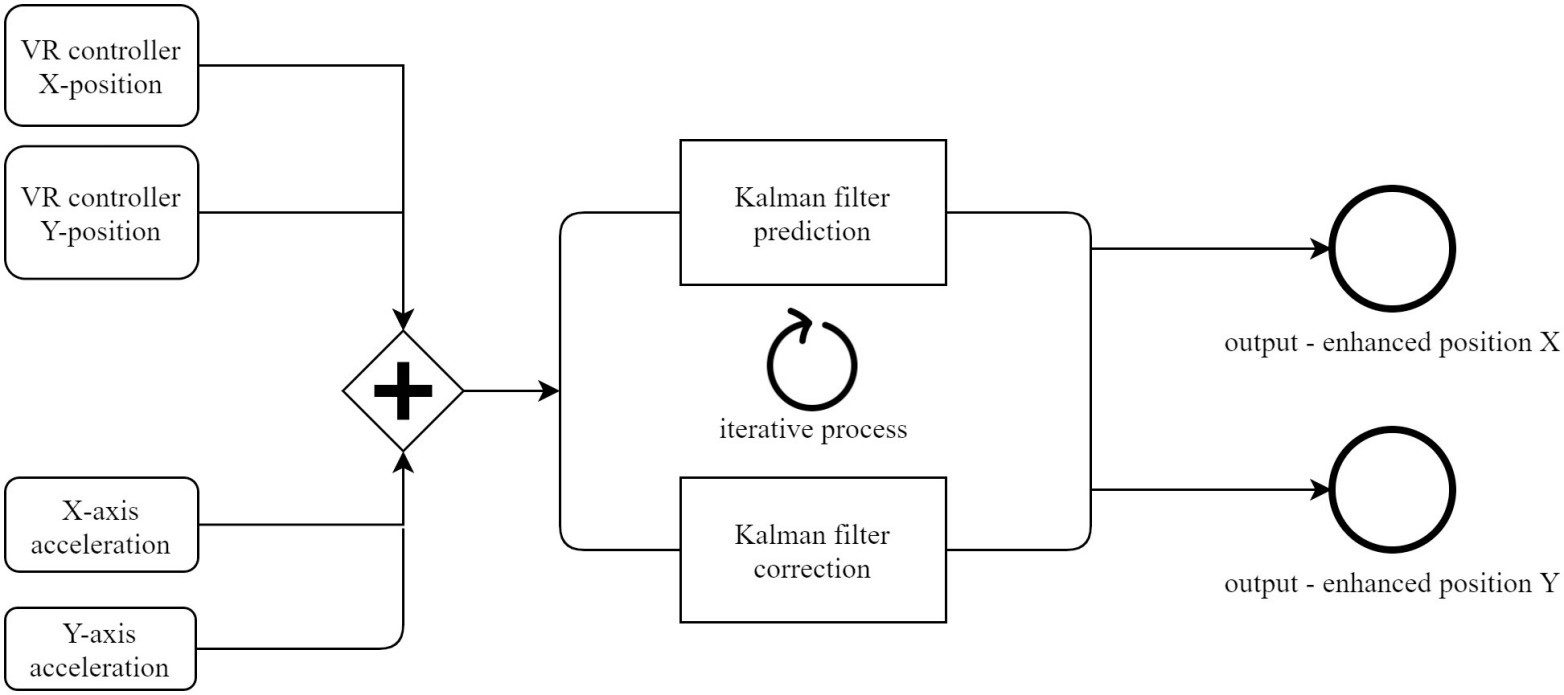
KF iteration process. The presented system merges position and acceleration data into a KF loop. The KF algorithm uses recursive methods for accurate estimations. The prediction and correction processes store and update the KF variables in the cycle.

Multi-sensor fusion of data is essential to improve the accuracy and recognition capabilities of the target tracking. We chose the KF algorithm in this study to cope with many applications in medical robotics ranging from perception to estimation of position and control. This is possible because the problem was approached with an augmented state vector in 6-D and in state space [15]. The KF consists of two processes that are aimed at appropriate estimation: prediction and correction. Fig 4 shows the implementation of the KF method in this study.

After gathering state data from the two sources, all of the estimates are used in the prediction phase to determine the next state and are iteratively updated in the correction process. The most crucial KF variables that provide a reliable estimate are sensor noise covariance and system noise variance from the correction step in Fig 4. It is not particularly easy to determine the most crucial KF variables for dynamic applications because the noise is random over time, thus determining them is not evident. The sensors incorporated in this analysis are the Oculus Touch VR controller and the IMU 9 DOF, respectively. The Oculus Touch gives information about the position and the IMU 9 DOF sensor provides acceleration data.

### System model for Kalman filter

A mathematical model of the presented system is required to implement the KF algorithm. The system model represents a mathematical expression which merges the position and acceleration from the VR controller and IMU sensor, respectively. We chose classical kinematic equations that integrate variables such as position, velocity, acceleration over time [16]. For the simplicity of the problem, we cast the kinematic equations into a state-space representation as follows:

$$\begin{array}{c} x_{k}=[\begin{array}{cccccc} 1 & 0 & 0 & \Delta t & 0 & 0\\ 0 & 1 & 0 & 0 & \Delta t & 0\\ 0 & 0 & 1 & 0 & 0 & \Delta t\\ 0 & 0 & 0 & 1 & 0 & 0\\ 0 & 0 & 0 & 0 & 1 & 0\\ 0 & 0 & 0 & 0 & 0 & 1 \end{array}]\cdot x_{k-1}+[\begin{array}{ccc} \frac{\Delta t_{}^{2}}{2} & 0 & 0\\ 0 & \frac{\Delta t_{}^{2}}{2} & 0\\ 0 & 0 & \frac{\Delta t_{}^{2}}{2}\\ \Delta t & 0 & 0\\ 0 & \Delta t & 0\\ 0 & 0 & \Delta t \end{array}]\cdot a_{k}, \end{array}$$ (1)

where the 6-D state vector **x_k_** represents the position (*p*) and velocity (*v*) of the forcep, [*p_x,k_p_y,k_p_z,k_v_x,k_v_y,k_v_z,k_*]^*T*^ at **k- th** time step, *Δt* is the time that the KF performs one iteration and the 3-D vector *a*_*k*_ is the acceleration data coming from the IMU sensor, [*ax*, *kay*, *kaz*, *k*]^*T*^. The state space model shows the relationship between input and state that can be implemented in the KF algorithm for an accurate estimation.

## Experiments

DGIST Institutional Review Board with research management number DGIST-20210608-HR-118–01 approved the study and all participants provided oral consent in the study. We designed a position matching task which was divided into two different conditions:

- Without the KF: millimeter scale VR environment with the KF deactivated.
- With the KF: millimeter scale VR environment with the KF activated.

**Fig 5:**
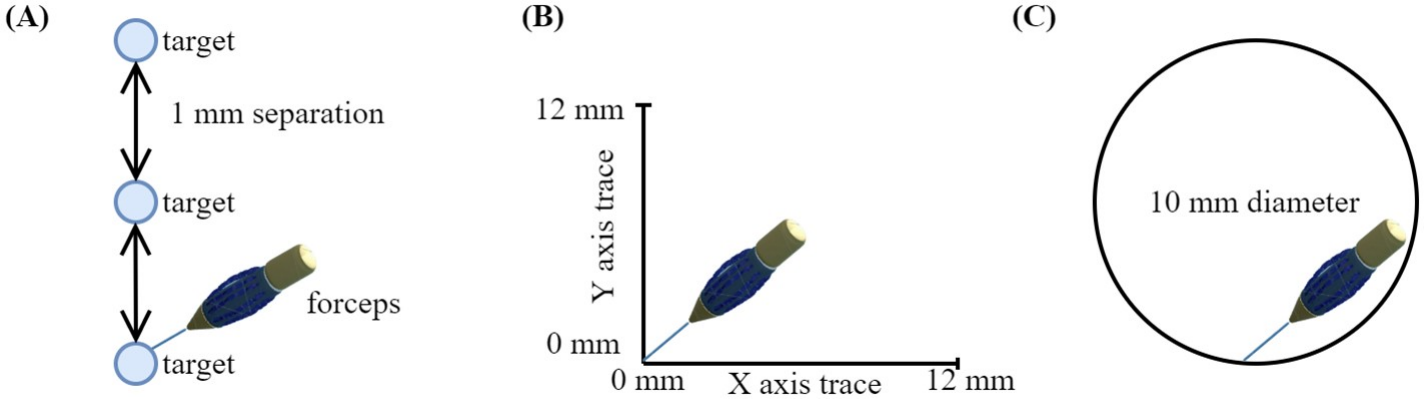
Experimental environments. (A) Point to point movement, where the distance between each target was 1 mm; (B) motorized trace, where the XY stage simulated a 12 mm trace along its axis of motion; and (C) 2-D trace, where the subjects traced a 10 mm diameter circle for the human experiments.

The millimeter scale VR environment always ran along the experiments under the influence of the KF. The program was capable of collecting both deactivated KF and activated KF data at the same time. We completed human/non-human experiments with the above-mentioned conditions to demonstrate the KF performance in the millimeter scale VR environment, as shown in Fig 5:

- Point to point movement
- Motorized trace
- 2-D axis trace

### Point to point movement

We asked the subject to stay in one position within the millimeter scale VR environment for 11 seconds in three different locations along the Y-axis. Fig 5A shows the distribution of points along with the VR space. A separation of 1 mm was set for each target.

### Motorized trace

We mounted the forceps on a linear motor stage, as shown in Fig 2. The linear motor pushed and moved the stage through the X and Y axes to 12 mm distance, as shown in Fig 5B. The reference trace was acquired from the linear motion stage. The linear motor had accurate and controlled motions. Additionally, the stage moved the forceps a distance of 12 mm along the X and Y axes. A trapezoidal motion profile was used with the stage in which the velocity was carried by a constant acceleration to the maximum velocity, and the motor traveled through a defined distance. The resolution of the linear motor was 29 μm.

### 2-D axis trace movement

**Fig 6:**
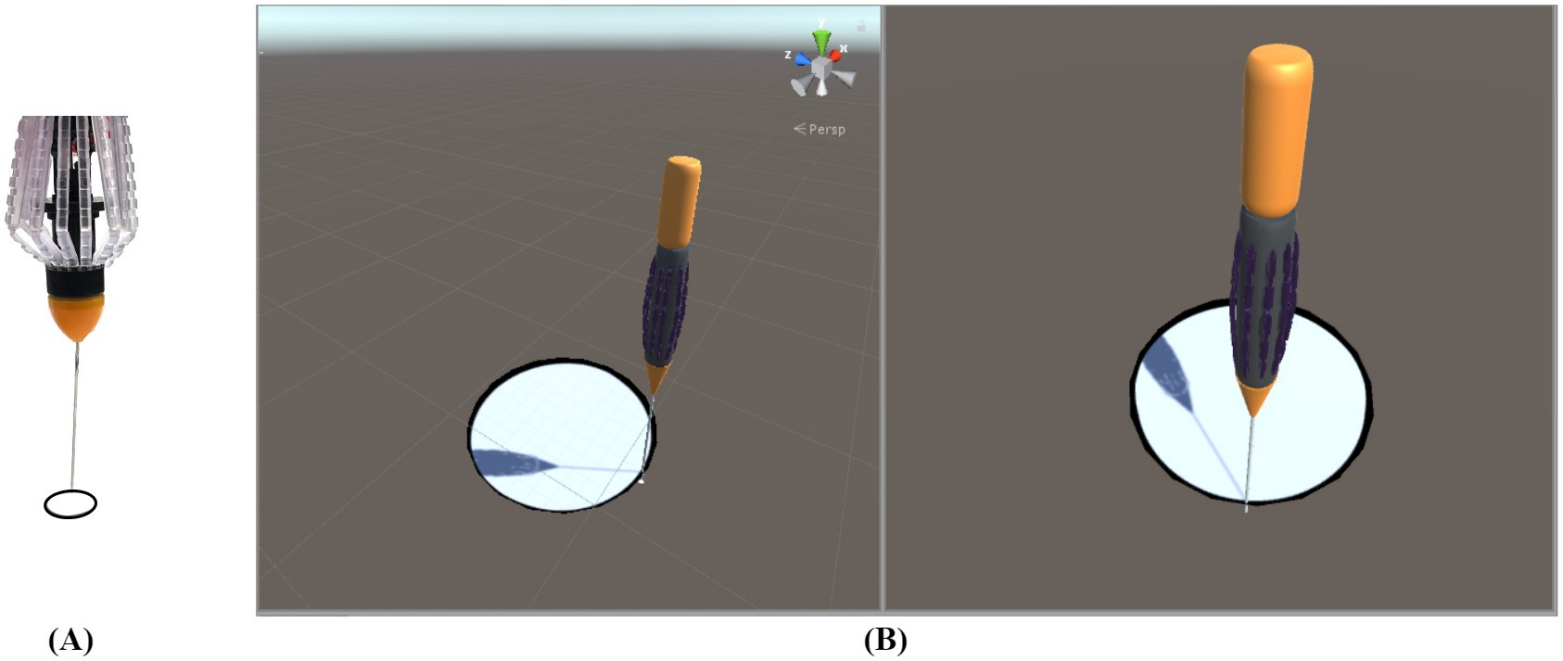
2-D trace. (A) Printed circle (B) VR circle 2-D trace. (A) shows a printed trace on a sheet and (B) represents a VR capture of a 2-D trace.

Fifteen subjects (13 males and 2 females. Mean age (standard deviation): 28.2 (± 2.5) years, range: 25–32 years) performed the trace of a 10 mm circle in Fig 5C. Additionally, the final dataset included the following distribution of participants in terms of VR experience: 3 participants (≥15 hours), 3 participants (5–14 hours), 6 participants (≤5 hours), and 3 participants did not have any experience. We divided all participants in two groups (A: six participants with VR experience of ≥15 hours and 5–14 hours. B: nine participants with VR experience of ≤5 hours and none experience). For this experiment, two different scenarios were implemented as shown in Fig 6:

- Printed circle: Every subject followed a trace of a 10 mm diameter circle printed on a sheet of paper, as shown in Fig 6A.
- VR circle: A virtual representation of a 10 mm diameter circle was designed for the VR scene. The subjects traced and followed the circle in the millimeter scale VR environment, as shown in Fig 6B.

We asked fifteen healthy subjects to trace the border of the circle with the forceps in the printed circle and VR circle scenarios.

#### Printed circle

**Fig 7:**
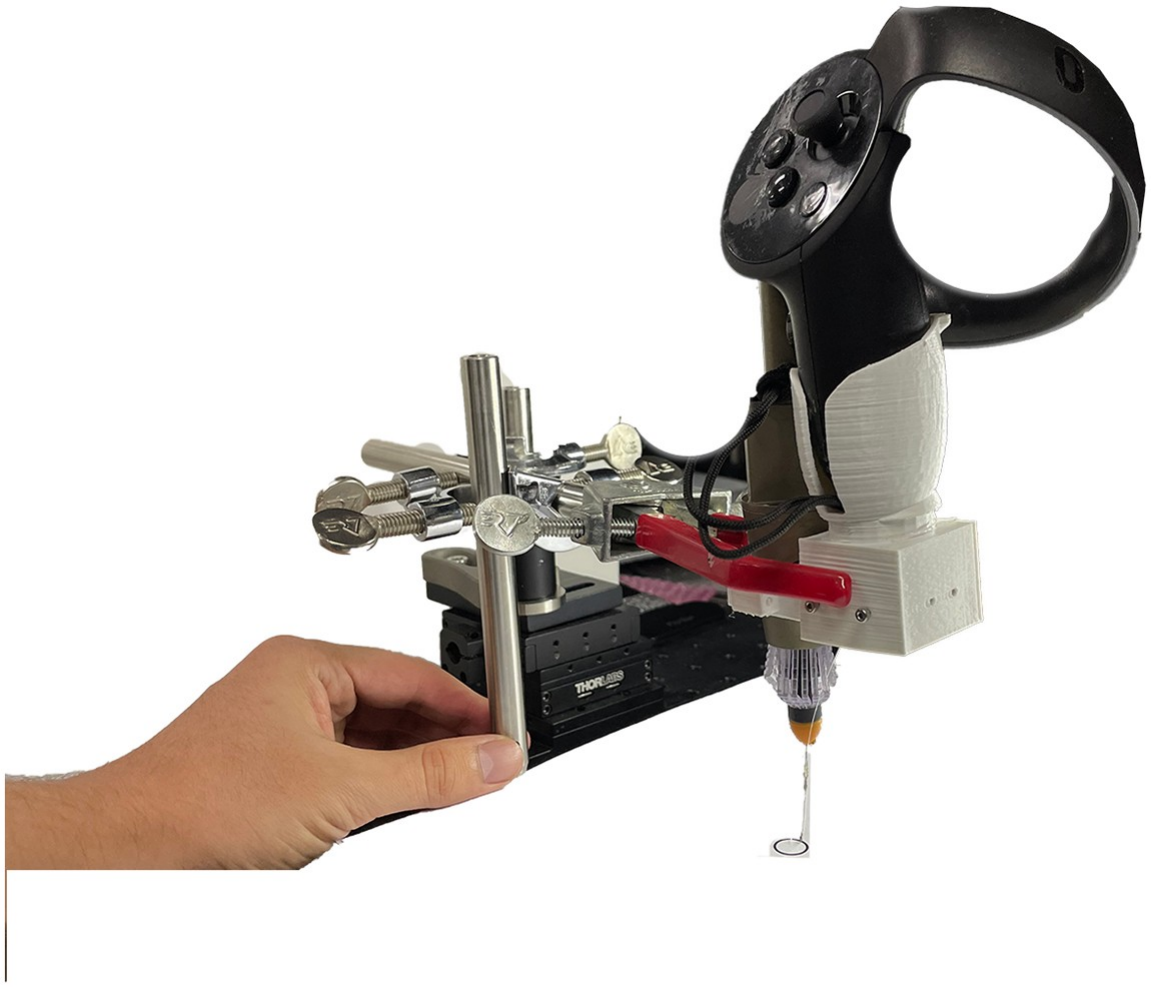
Subject performing the 2-D printed circle experiment. The figure shows one subject conducting the experiment with manual movement of the linear stage. A 10 mm circle is printed on a sheet of paper. The subject traced the circle by using the linear stage manually.

Each subject followed the edge of the printed circle without using the HMD. They were able to move the forceps manually by using the linear stage. Fig 7 shows one subject following the printed circle edge without the HMD.

#### VR circle

Each subject followed the edge of a VR circle while wearing the HMD by using the linear stage. The subjects moved the forceps fixed on the main axis.

## Results and discussion

### The point to point movement

**Fig 8:**
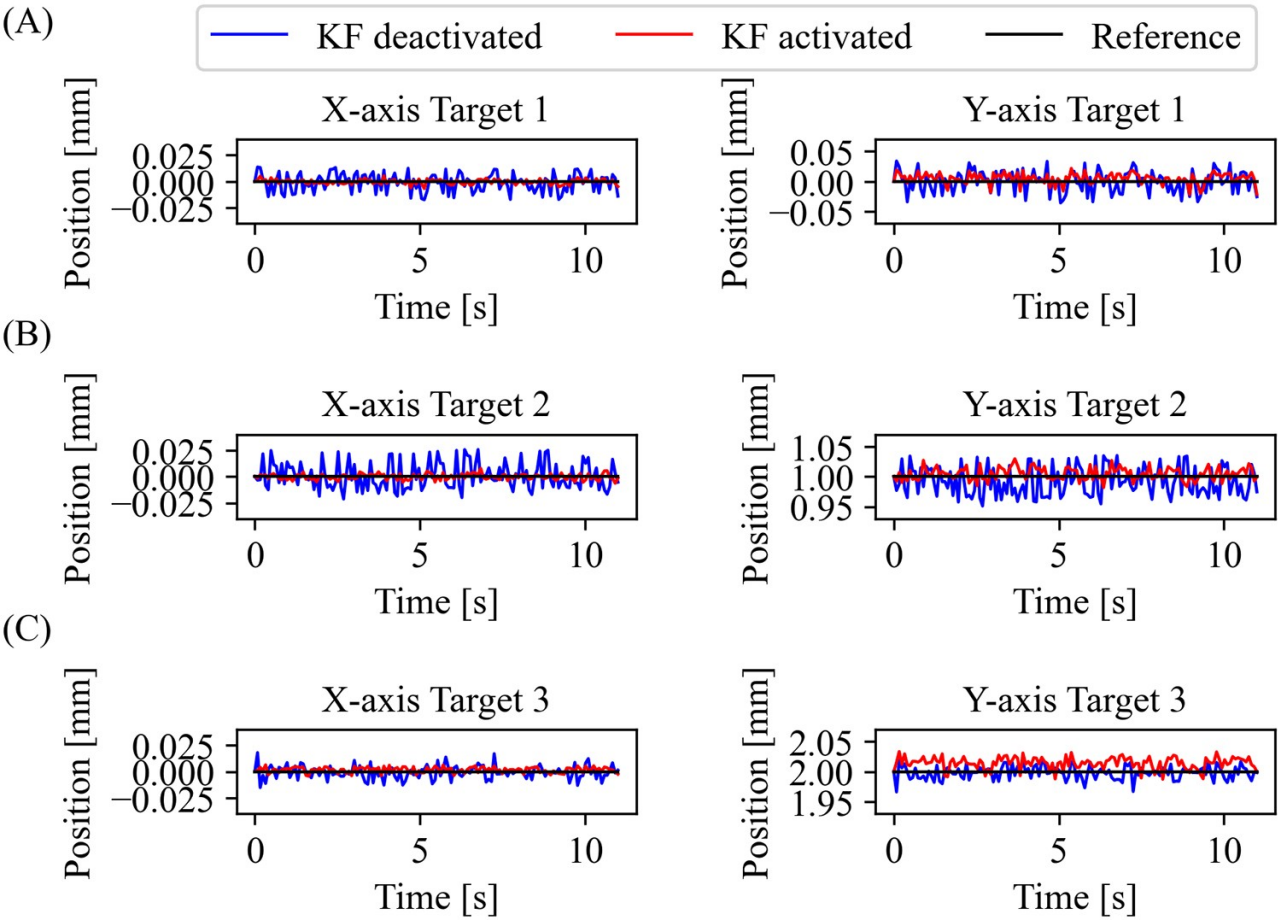
Experimental results of point to point movement. One subject attempted to place the forceps in three different reference points (black). In each trial, the subject stayed still for 11 seconds under the KF deactivated (blue) and activated (red). (A) Target 1 location (mm): X = 0 Y = 0, (B) Target 2 location (mm): X = 0 Y = 1, and (C) Target 3 location (mm): X = 0 Y = 2.

We asked the subject to place the forceps in three different locations along the Y axis for 11 seconds. Fig 8 shows the performance of the KF in a stationary point to point movement task. Deviation from the reference is noticeable at every point with the KF deactivated. The implementation of the KF makes the system follow the reference more accurately as seen in Fig 8A–8C.

The results show a tendency to be accurate in following the reference when the KF is activated. The improvement of the system is more than 43% in most of the cases as shown in Table 1. We performed a paired t–test with a significant threshold of p < 0.05 between KF deactivated and KF activated conditions. We found p-values of 0.009 and 0.016 for the X and Y axes, respectively. The KF activated mode significantly improved the system in a static situation.

**Table 1:** The RMSE of point to point movement results.

Target	RMSE result (mm)					
	KF deactivated		KF activated		Improvement (%)	
	X	Y	X	Y	X	Y
1	0.0168	0.0380	0.0027	0.0166	83.93	56.32
2	0.0134	0.0283	0.0029	0.0159	78.36	43.82
3	0.0196	0.0338	0.0026	0.0100	86.73	70.41

### Motorized trace result

**Fig 9:**
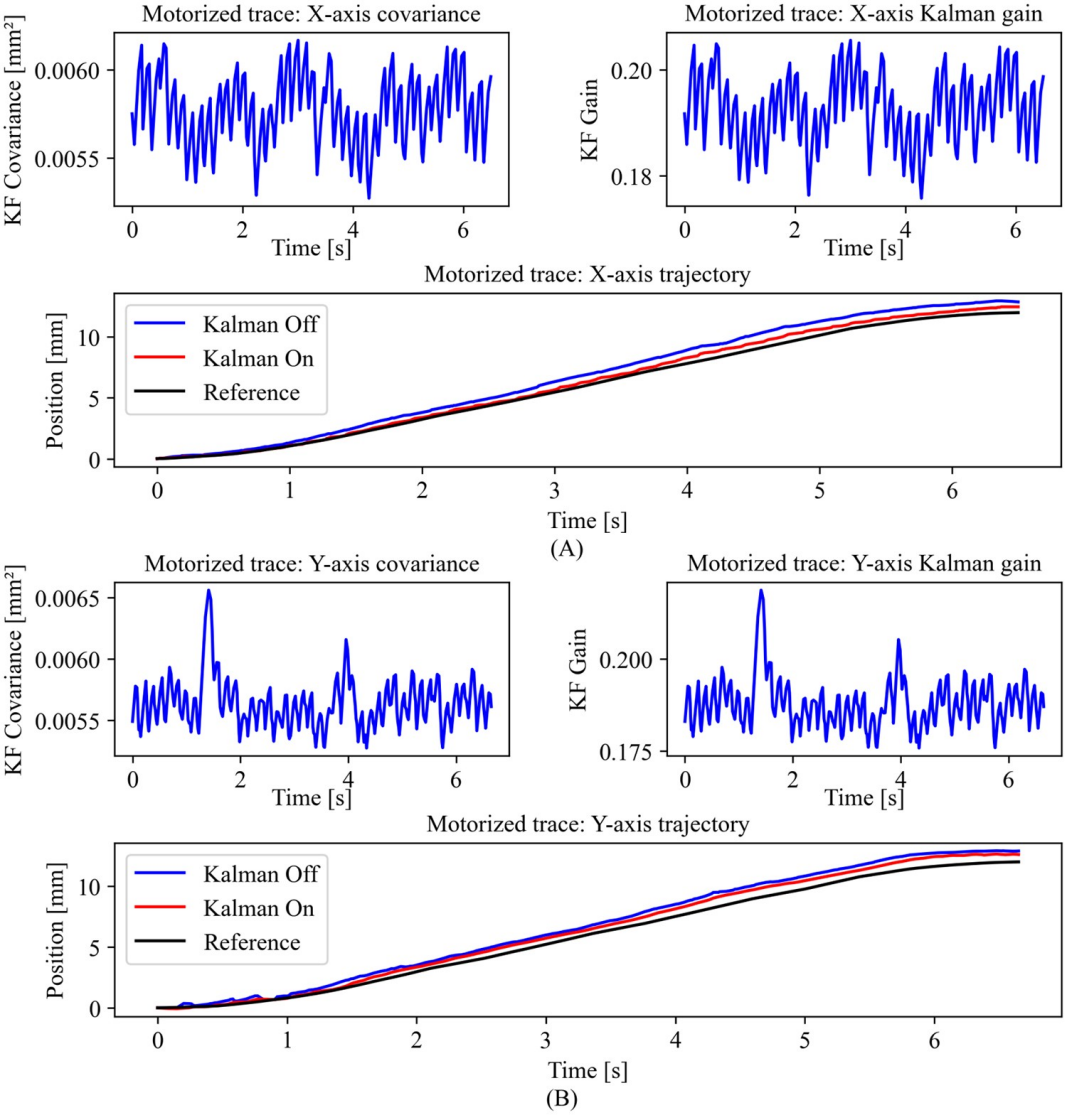
Motorized trace result. Covariance, Kalman gain, and position over time for the X-axis (A) and Y-axis (B) are shown. The covariance indicates the accuracy of the state calculation along the X-axis (A) and Y-axis (B) motion. The predicted position (red) is compared under two conditions: when the KF is deactivated (blue) and the reference (black). In the KF algorithm, the Kalman gain is updated. Hence, the Kalman gain adapts to the motion along the X-axis (A) and Y-axis (B).

In Fig 9, the error covariance graph shows how accurately the state estimates in the X-axis (Fig 9A) and the Y-axis (Fig 9B). The Kalman gain is updated along the time in the KF algorithm. The accuracy is notable when the KF is activated, and the deviation is high from the starting point of the task without KF. Notice that the filter quickly adapts to the reference value in both cases (X and Y axis). The Kalman gain acts as a regulator between the estimate and the measurement. Trace precision is evident when the KF is enabled. The system lacks accuracy when the KF is deactivated. Moreover, a high deviation of the trace is critically noticeable. We performed a t-test with a level of significance of p < 0.01 between the KF disabled and enabled. We obtained p-values of p < 0.001 in both X and Y axes. The KF activated shows a significant improvement in a dynamic scenario.

### 2-D axis trace result

#### Printed circle result

**Fig 10:**
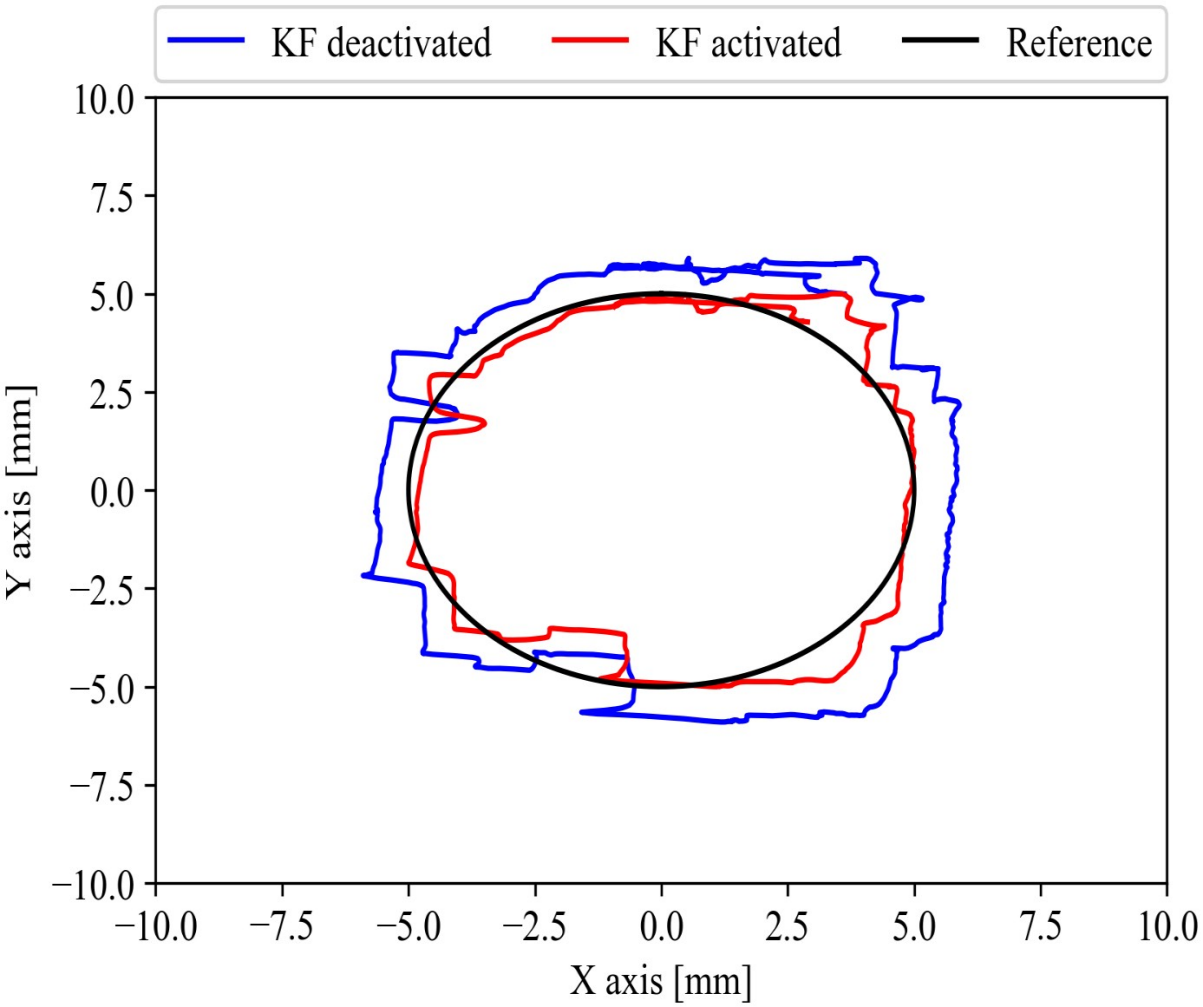
One subject 2-D axis trace (printed circle) result. The subject without the HMD performed the circle trace. The KF activated (red) shows a tendency to be more accurate than the KF deactivated (blue) along the reference (black).

The results in Fig 10 show the trace performance by one of the subjects while performing the 2-D printed circle experiment with one hand. In addition, the Fig 10 shows the 2-D positions of the trace with the KF activated and deactivated conditions of the subjects along the 10 mm circle (reference). The subjects’ traces had an arbitrary starting point. Each subject took their own time to start and finish the task. The KF activated algorithm more precisely followed the reference. The results of the KF deactivated always showed a considerable deviation compared with the KF activated. In addition, the KF deactivated and activated conditions were operated at the same time while the subjects were participating in the experiment.

In Table 2, we used one-tailed paired t-tests with a significance level of p-value < 0.01 within the group A (six participants with VR experience of ≥15 hours and 5–14 hours) and B (nine participants with VR experience of ≤5 hours and none experience). We calculated the average RMSE for the 2-D printed circle trace under sensor fusion activated and sensor fusion deactivated conditions. For group A, we found a significant difference in the average RMSE while KF was deactivated (M = 1.1307 mm, SD = 0.2307 mm) and activated (M = 0.8993 mm, SD = 0.2361 mm) with p = 0.001. Similarly, in group B, the average RMSE with KF deactivated (M = 0.9953 mm, SD = 0.2953 mm) and activated (M = 0.7409 mm, SD = 0.3398 mm) showed a significant difference with p < 0.001. The average RMSE improvements in groups A and B were 20.69% ± 8.41% and 28.36% ± 18.31%, respectively. The participants in group B showed better performance in comparison to the participants in group A, even under the KF deactivated mode. The KF activated mode significantly improved the performance in both groups. The intrinsic hand tremor might have a significant role in the results.

**Table 2:** T-test results of printed circle experiment.

Group	Average RMSE [mm]		Average RMSE improvement [%]	P-value
	KF deactivated	KF activated		
A	1.1307 ± 0.2307	0.8993 ± 0.2361	20.69 ± 8.41	0.001
B	0.9953 ± 0.2953	0.7409 ± 0.3398	28.36 ± 18.31	<0.001

#### VR circle result

**Fig 11:**
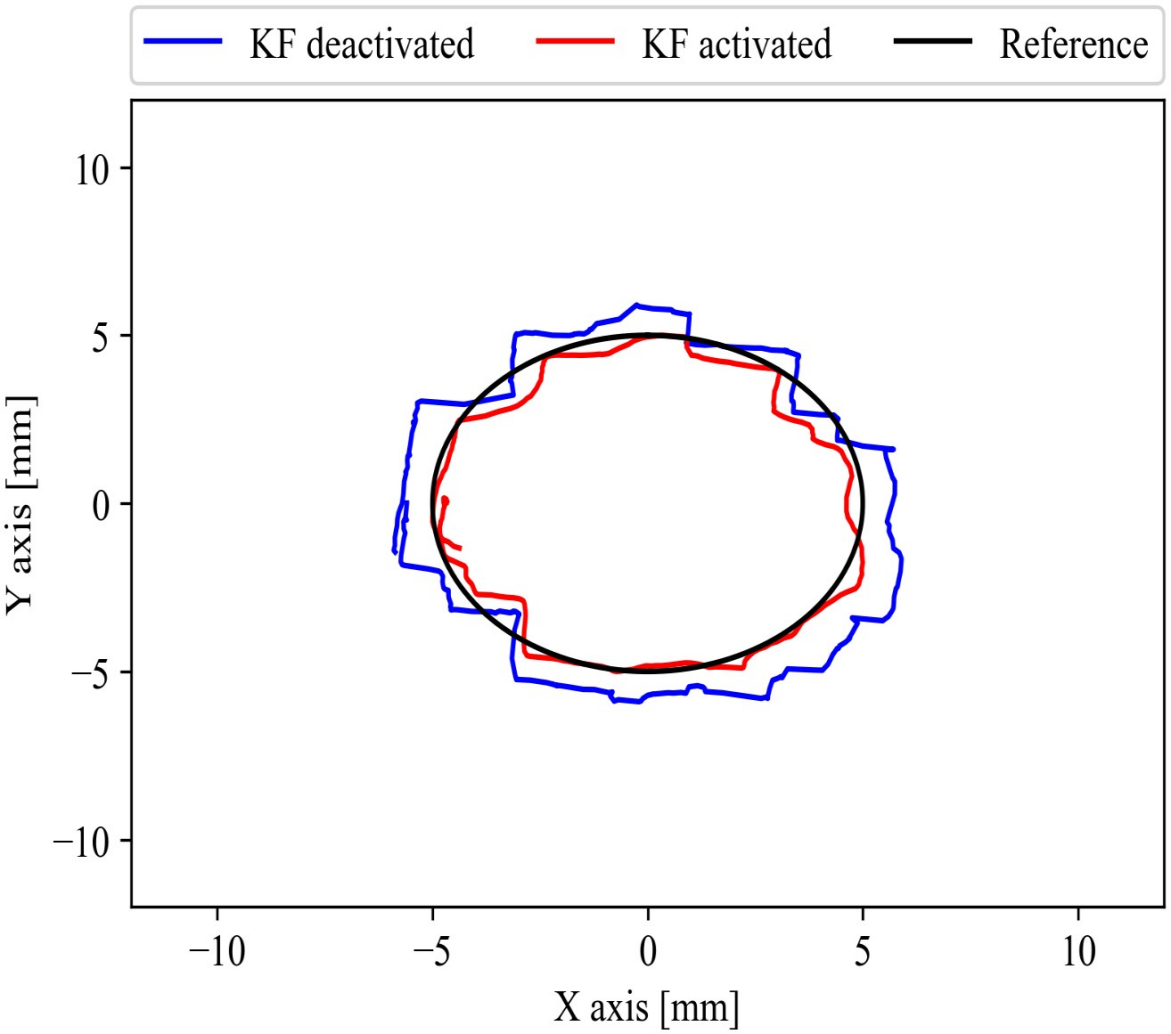
One subject 2-D axis trace (VR circle) result. The subject with the HMD showed more tendency to follow the reference (black) with KF activated (red) than under KF deactivated (blue).

Fifteen subjects followed and traced the edge of a VR-2-D 10 mm diameter circle displayed in the HMD. With the help of a stage, the subjects moved the forceps fixed on one axis. Fig 11 shows the performance from one of the fifteen subjects in the millimeter scale VR environment while following the edge of the VR circle. Each subject wore the HMD in which a 2-D virtual circle was displayed through the HMD. Each subject had the opportunity to practice before the experiment started, and so became accustomed to the millimeter scale VR environment.

Table 3 shows the one-tailed paired t-test results with a significance threshold of p-value = 0.01 within the group A (six participants with VR experience of ≥15 hours and 5–14 hours) and B (nine participants with VR experience of ≤5 hours and none experience). We compared the average RMSE between KF activated and deactivated for the VR circle trace experiment. During the experiment, the subjects were operating in the millimeter scale VR environment under the KF activated parameters. For participants in group A, we found a significant decrease in the average RMSE of KF activated (M = 0.7576 mm, SD = 0.1528 mm) in comparison to KF deactivated (M = 0.9983 mm, SD = 0.3061 mm) with p = 0.006. Also, the group B showed a significance difference in the average RMSE of KF deactivated (M = 1.0864 mm, SD = 0.2460 mm) in comparison to KF activated (M = 0.7944 mm, SD = 0.1970 mm) with p < 0.001. The average RMSE improvement was higher in the group B (M = 26.58%, SD = 5.84%) in comparison to group A (M = 21.65%, SD = 10.63%).

**Table 3:** T-test results of VR circle experiment.

Group	Average RMSE [mm]		Average RMSE improvement [%]	P-value
	KF deactivated	KF activated		
A	0.9983 ± 0.3061	0.7576 ± 0.1528	21.65 ± 10.63	0.006
B	1.0864 ± 0.2460	0.7944 ± 0.1970	26.58 ± 5.84	<0.001

In addition, we compared the average RMSE improvement between printed (Table 2) and VR circle (Table 3) results. The experienced participants (group A) had a higher average RMSE improvement in the VR circle (M = 21.65%, SD = 10.63%) in comparison to printed circle (M = 20.69%, SD = 8.41). On the other hand, the group B showed higher average RMSE improvement in the printed circle (M = 28.36%, SD = 18.31%) in comparison to the VR circle (M = 26.58%, SD = 5.84%). Also, both groups showed a high reduction of the average RMSE results with KF activated in comparison to results with KF deactivated in both scenarios. In general, experienced and novice VR participants showed a significant improvement in performance when the VR and KF modes were enabled.

The precision of the trace of some subjects was more noticeable thanks to the visual stimuli that the HMD executes on the human eye. Some subjects followed the edge more accurately than the one in the printed circle scenario. The perspective that the VR world offered to the subjects enabled them to achieve a better performance compared with the 2-D printed circle case. The VR world can be explored interactively at a workstation, usually by manipulating a VR controller with KF. Hence, the tracking performance with the VR world was more effective in all participants. The implemented VR system accelerates a positioning task performance particularly in VR experienced subjects. The VR controller and KF are crucial in small scale VR scenarios. This approach highly reduced the positioning error of a commercial controller in both dynamic and static scenarios.

## Conclusion

The efficacy of a sensor fusion, KF algorithm was proved in a C# real-time application based on a millimeter scale VR technology. The output from the sensor fusion algorithm showed high improvements compared with a traditional VR tracking system. The experiments conducted in this study demonstrated a potential increase of accuracy in both the X and Y-axis for a variety of micromanipulation tasks. The KF algorithm was validated in both stationary and dynamic situations. In printed circle experiment with KF activated, the highest improvement of tracking was 58.27% with a RMSE of 0.2883 mm. Experienced and novice VR subjects demonstrated a meaningful enhancement in micromanipulation performance with the VR and KF modes activated in the millimeter scale VR space. In future work, we will test various sensor fusion algorithms to maximize micromanipulation performance more clearly in a millimeter scale VR training system. Moreover, by combining VR with sensor fusion technologies, various kind of VR scenarios can be beneficial to train future microsurgeons including state-of-art microsurgical systems [17–20].

## Supporting information

S1 File (ZIP)
